# Racial differences in quantitative optical coherence tomography angiography findings between older non-diabetics with co-morbidities

**DOI:** 10.1371/journal.pone.0285360

**Published:** 2023-05-05

**Authors:** John Moir, Sarah H. Rodriguez, Lindsay Y. Chun, Nathalie Massamba, Dimitra Skondra

**Affiliations:** 1 Pritzker School of Medicine, University of Chicago, Chicago, Illinois, United States of America; 2 Department of Ophthalmology and Visual Science, University of Chicago, Chicago, Illinois, United States of America; 3 Department of Ophthalmology, Handicap, and Vision, Pitie Salpetriere Hospital, Sorbonne University, Paris, France; 4 J. Terry Ernest Ocular Imaging Center, University of Chicago, Chicago, Illinois, United States of America; University of California Los Angeles, UNITED STATES

## Abstract

This cross-sectional study compared optical coherence tomography angiography (OCTA) parameters between older Black and White adults with systemic comorbidities in an effort to further understand racial differences in the retinal microvasculature. We analyzed vessel density at the superficial (SCP), intermediate (ICP), and deep capillary plexuses (DCP), foveal avascular zone (FAZ) parameters, and blood flow area (BFA) at the choriocapillaris. We used a mixed-effects linear regression model, controlling for hypertension and two eyes from the same subject, to compare OCTA parameters. Black subjects had lower foveal vessel density at the SCP and ICP, while no differences were observed at the parafovea or 3x3 mm macular area of any capillary layer. Black subjects had greater FAZ area, perimeter, and FD-300, a measurement of vessel density in a 300 μm wide ring around the FAZ. Black subjects also had lower BFA at the choriocapillaris. Within a cohort of subjects without hypertension, these differences remained statistically significant, with the exception of foveal vessel density at the SCP and foveal BFA of the choriocapillaris. These findings suggest that normative databases of OCTA parameters must strive to be diverse in nature to adequately capture differences across patient populations. Further study is required to understand if baseline differences in OCTA parameters contribute to epidemiological disparities in ocular diseases.

## Introduction

Optical coherence tomography angiography (OCTA) is a novel, non-invasive imaging method that provides three-dimensional visualization of blood flow within the retinal microvasculature [1]. This enables the quantitative analysis of OCTA parameters such as vessel density (VD), foveal avascular zone (FAZ), and % blood flow area (BFA), which traditional imaging techniques, like fluorescein angiography, don’t provide due to poorer imaging of the deep capillary plexus (DCP) and choroidal vasculature [2]. Furthermore, the recent introduction of commercial projection artifact removal (PAR) software has facilitated the visualization and quantification of the three distinct capillary plexuses of the retina [3,4]. With these advantages, it comes as no surprise that OCTA has been used to study a wide variety of ocular diseases. Diabetic retinopathy, retinitis pigmentosa, retinal vein occlusion, and glaucoma are well-covered throughout the literature, with OCTA generally demonstrating impaired vascular flow in eyes with these conditions as compared to healthy controls [5–9].

Many studies that have reported normative OCTA databases of healthy individuals are either represented overwhelmingly by patients of Caucasian ancestry with relatively few minorities included or fail to describe the racial and ethnic backgrounds of enrolled patients [10–14]. Our group has previously found that compared to young healthy White adults, young healthy Black adults have significantly lower VD at the superficial (SCP) and intermediate capillary plexuses (ICP) [15]. We’ve also found that young healthy Black adults have a greater FAZ area and lower BFA of the choriocapillaris [15,16]. Variations in OCTA parameters by race or ethnicity is a topic that is relatively unexplored at the present moment. However, it merits further consideration to better understand the impact of ocular diseases across diverse patient groups.

While our previous studies focused on young adults at an optimal state of health, we chose to focus on older adults herein, a patient population that has not been extensively studied in this context. We aim to compare VD at the three capillary plexuses, FAZ, and BFA at the choriocapillaris in an effort to further understand differences in OCTA parameters between Black and White adults.

## Design and methods

### Study design

This cross-sectional study was approved by the Institutional Review Board of the University of Chicago (IRB #18–1174). Study protocols adhered to the tenets of the Declaration of Helsinki and conformed to the Health Insurance Portability and Accountability Act regulations. The study was conducted between September 2017 and August 2021. All subjects provided written informed consent.

### Study participants

This cross-sectional study was conducted at the University of Chicago Medical Center’s Ophthalmology Clinic. Inclusion criteria were non-Hispanic Black and non-Hispanic White non-diabetics. Patients with diabetes mellitus, diabetic retinopathy, other retinal vasculature diseases, glaucoma, uveitis, visually significant cataracts graded above nuclear opalescence or nuclear grade three, astigmatism greater than 3 diopters (D), myopia greater than 5 D, or hyperopia greater than 3 D were excluded from the study. Subjects were prospectively consented for OCTA imaging for further retinal evaluation. Chart review was performed for additional demographic, medical, and ocular information. Systemic diseases, including hypertension, hyperlipidemia, smoking status (never or former/current), and heart disease (history of myocardial infarction, heart failure, and/or coronary artery disease) were recorded as binary variables. Smoking pack-years at the time of imaging were calculated for each former and current smoker. Estimated glomerular filtration rate (GFR) was recorded for each patient to track kidney functioning. Subjects who self-identified with races other than “White” or “Black” were excluded from the analysis.

### OCTA image acquisition

**Fig 1:**
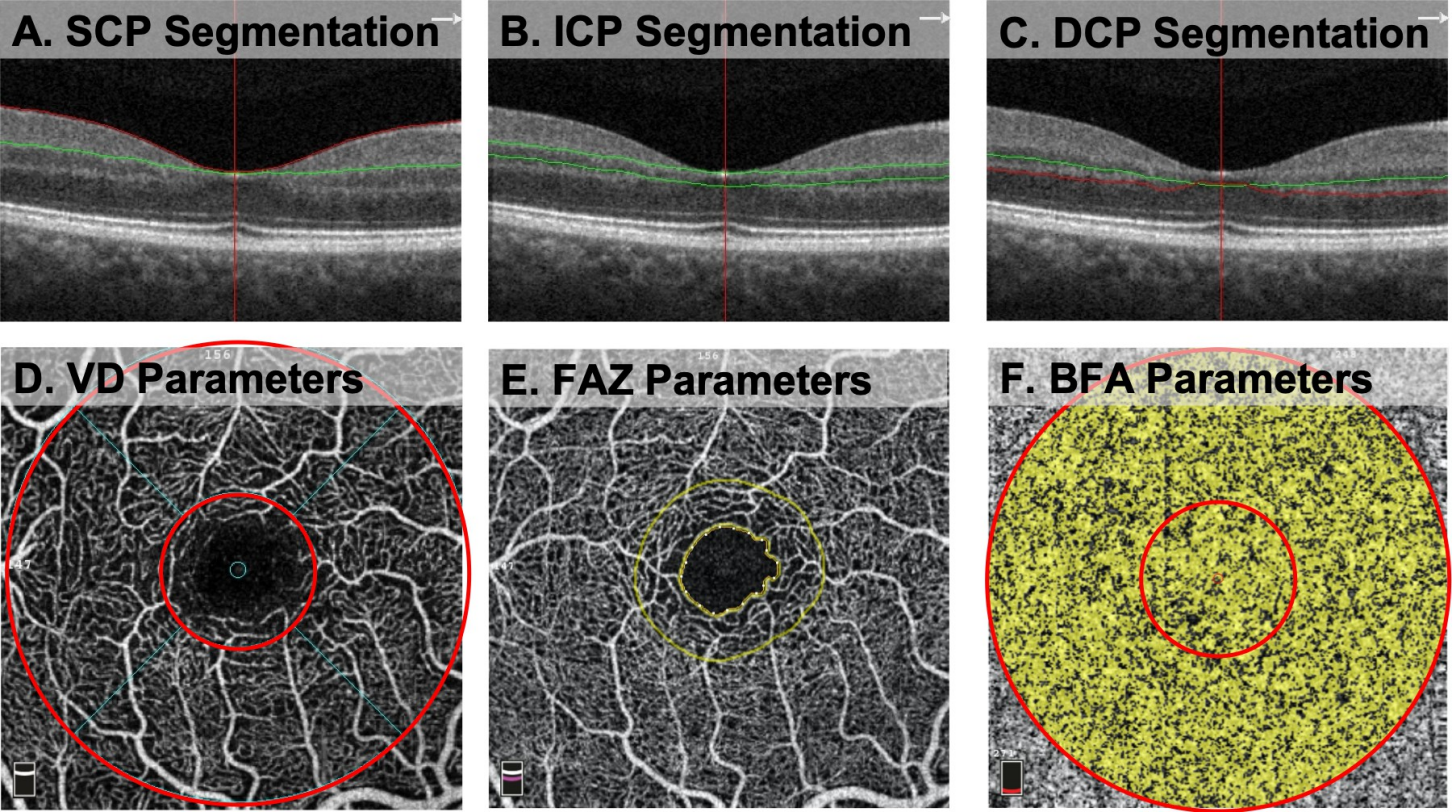
Optical coherence tomography angiography (OCTA) segmentation and quantitative analysis of OCTA Parameters. (A-C) Segmentation boundaries for the superficial (SCP), intermediate (ICP), and deep capillary plexuses (DCP) shown on B-Scan. Red and green lines on B-scan represent segmentation boundaries of each plexus. (D) *En face* OCTA image of vessel density (VD) calculation. Inner ring (1mm diameter) represents the fovea and outer ring (3mm diameter) represents the 3x3 mm macular area. Area between the inner and outer ring represents the parafovea. (E) Measurement of the foveal avascular zone (FAZ) area using built-in AngioAnalytics software. Outer circle represents the FD-300 VD, a measurement of VD in a 300 μm ring around the FAZ for the full retinal thickness. (F) *En face* OCTA image of the choriocapillaris blood flow area (BFA) with inner ring (1mm diameter) representing the fovea and outer ring (3mm diameter) representing the 3x3 mm macular area. Area between the inner and outer ring represents the BFA of the parafovea.

Images were obtained using the Optovue RTVue XR Avanti (Optovue Inc, Fremont, California, USA Version 2018.1.8.63) with phase 7 AngioVue software. This machine has an A-scan rate of 70,000 scans per second. Images were taken using a 840 nm light source and a 45 nm bandwidth. Two consecutive B-scans, composed of 304 A-scans each, were acquired in a 3x3 mm^2^ region centered on the fovea. We used custom segmentation for the SCP, ICP, and DCP, as described by Nesper et al [17]. The SCP was bounded from the internal limiting membrane (ILM) to 10 um above the IPL (Fig 1A). The ICP was bounded from 10 um above to 30 um below the IPL (Fig 1B). The DCP was bounded from 30 um below the IPL to 10 um below the OPL (Fig 1C). Each image was manually checked for errors in segmentation, which were excluded if found to exist. Additional prerequisites included OCTA images without significant movement or shadow artifacts, and a signal strength index (SSI) greater than 55 (manufacturer’s recommendation).

### Quantitative analysis of vessel density and FAZ

To generate a vessel density measurement at the SCP, ICP, and DCP, AngioAnalytics software calculated the percentage of the image occupied by blood vessels. An annulus centered on the fovea was overlaid on the 3x3 mm macular scan. The annulus was composed of two concentric circles of 1 mm and 3 mm in diameter (Fig 1D). The fovea was defined as the inner 1 mm circle and the parafovea was defined as the area between the inner 1mm and outer 3 mm circles. The 3x3 macular area was the combined fovea and parafovea. The FAZ area was measured using built-in software and represented the area (mm^2^) surrounding the fovea devoid of retinal capillaries (Fig 1E). The FAZ perimeter represented the length of the outline around the FAZ. The acircularity index (AI) was defined as the ratio of the perimeter of the FAZ to the perimeter of a circle with equal area. The vascular density in a ring with a 300 μm width surrounding the FAZ from the ILM to the OPL (FD-300 VD) was calculated as the percentage of pixels in the ring occupied by blood vessels.

### Quantitative analysis of blood flow area at the choriocapillaris

AngioAnalytics automatically calculated BFA, which represents the percentage of the image occupied by blood vessels. This measurement was obtained at the level of the choriocapillaris for the circular areas of 1 mm and 3 mm diameter manually centered on the fovea (Fig 1F). The central 1 mm circle represented the BFA of the fovea. BFA of the parafovea was calculated by subtracting the BFA of the 1 mm circle from the BFA of the 3 mm circle.

### Statistical analysis

Statistical analysis was performed using Stata16.1 (StataCorp LP, College Station, TX). For baseline demographic data, we used Fisher’s exact test to calculate P values of categorical variables and Wilcoxon rank-sum tests to calculate P values of continuous variables. All data is presented as mean values with standard deviations. Frequency weighting was used to account for correlation between two eyes of the same subject. A mixed-effects linear regression model, controlling for two eyes of the same patient and hypertension, was used to determine the association between race and OCTA parameters. OCTA parameters from a subgroup of patients without hypertension were analyzed with a mixed-effects linear regression model that controlled for two eyes from the same patient. A P value of <0.05 was considered statistically significant.

## Results

86 eyes from 57 subjects were included in this study. 32 eyes were excluded due to exclusion criteria being met in a single eye (signal strength < 55, ocular conditions affecting the retinal microvasculature unilaterally, motion artifacts present on OCTA scan, etc.). 27 subjects self-identified as Black (mean age 56.9±14.4 years, 26% male), and 30 subjects self-identified as White (mean age 51.7±16.1 years, 43% male). There were no significant baseline differences in the demographics and clinical characteristics of the two racial groups, other than a higher rate of diagnosed hypertension in Black subjects (44% in Black vs. 16% in White subjects, p = 0.03) (Table 1). There was no significant difference in the SSI (68.3±7.1 in Black vs. 67.5±8.8 in White subjects, p = 0.45). A subgroup of patients without hypertension also had no baseline differences with respect to all demographic and clinical characteristics (Table 1).

**Table 1:** Baseline patient demographics and clinical characteristics.

**Entire Cohort**	**White subjects**	**Black subjects**	**P-Value**
# Subjects (eyes)	30 (46)	27 (40)	
Sex (M:F)	13:17	7:20	0.26^a^
Age (years)	51.73 ± 16.10	56.89 ± 14.41	0.86^b^
Hypertension (%)	4 (13%)	12 (44%)	0.02^a^*
Hyperlipidemia (%)	5 (17%)	6 (22%)	0.74^a^
Heart Disease (%)	4 (13%)	3 (11%)	1^a^
Smoking status (never:former/current)	25:5	18:9	0.22^a^
Smoking (pack-years)	1.33 ± 4.75	3.72 ± 11.34	0.51^b^
GFR (mL/min)	84.93 ± 16.30	84.21 ± 21.86	0.59^b^
Signal Strength Index	67.53 ± 8.77	68.31 ± 7.10	0.45^b^
**Non-Hypertensive Subgroup**	**White subjects**	**Black subjects**	**P-Value**
# Subjects (eyes)	26 (39)	15 (22)	
Sex (M:F)	11:15	4:11	0.50^a^
Age (years)	51.29 ± 15.96	50.53 ± 17.41	0.90^b^
Hypertension (%)	0 (0%)	0 (0%)	1^a^
Hyperlipidemia (%)	3 (12%)	0 (0%)	0.29^a^
Heart Disease (%)	3 (12%)	2 (13%)	1^a^
Smoking status (never:former/current)	4:22	10:5	0.25^a^
Smoking (pack-years)	0.27 ± 1.03	5.92 ± 15.14	0.4^b^
GFR (mL/min)	84.2 ± 14.53	93.64 ± 27.74	0.62^b^
Signal Strength Index	67.79 ± 8.92	68.16 ± 7.49	0.65^b^

^a^Fisher’s exact and

^b^Wilcoxon rank-sum tests were used to calculate p-values.

*Significant at P < 0.05. GFR: Glomerular filtration rate.

Vessel density values across the three capillary plexuses are reported in Table 2. Within the SCP, Black subjects had significantly lower foveal VD (15.03 ± 7.54 vs. 21.07 ± 8.07, p = 0.02). There was no significant difference in parafoveal VD of the SCP or the VD of the 3x3 mm macular area of the SCP. Black subjects had significantly lower foveal VD within the ICP (28.03±8.19 vs. 35.44±6.51, p<0.001). There were no significant differences in the VD of the parafovea or 3x3 mm macular area within the ICP. No significant differences in VD were observed at the fovea, parafovea, or 3x3 mm macular area of the DCP. When these analyses were repeated in a subgroup of patients without hypertension, Black subjects had a significantly lower foveal VD at the ICP (30.95±8.14 vs. 35.52±6.84, p = 0.008). No other significant differences were noted at the ICP or DCP in patients without hypertension. However, there was a non-significant trend for Black subjects to have lower VD at the 3x3 mm macular area of the ICP (43.29 ± 3.50 vs. 44.17 ± 3.60, p = 0.053).

**Table 2:** Comparison of OCTA parameters between Black and White subjects.

Macular Capillary Parameter	All White Subjects	All Black Subjects	P-value^a^	Non-HTN White Subjects	Non-HTN Black Subjects	P-value^b^
**SCP VD (%)**						
3x3 mm macular area	45.74 ± 4.22	46.03 ± 4.41	0.28	46.02 ± 3.87	47.92 ± 3.40	0.33
Fovea	21.07 ± 8.07	15.03 ± 7.54	0.02*	20.93 ± 7.79	17.37 ± 8.01	0.13
Parafovea	48.49 ± 3.99	49.33 ± 4.41	0.09	48.79 ± 3.69	51.15 ± 3.44	0.15
**ICP VD (%)**						
3x3 mm macular area	43.74 ± 4.10	43.88 ± 4.30	0.42	44.17 ± 3.60	43.29 ± 3.50	0.053
Fovea	35.44 ± 6.51	28.03 ± 8.19	< 0.001*	35.52 ± 6.84	30.95 ± 8.14	0.008*
Parafovea	44.98 ± 4.66	45.77 ± 4.66	0.98	45.38 ± 4.13	44.93 ± 3.41	0.28
**DCP VD (%)**						
3x3 mm macular area	40.91 ± 10.75	42.60 ± 8.47	0.73	39.10 ± 10.19	43.03 ± 7.69	0.74
Fovea	25.32 ± 10.36	23.58 ± 8.96	0.33	23.21 ± 8.67	25.77 ± 9.8	0.76
Parafovea	43.11 ± 11.23	45.20 ± 8.77	0.59	41.33 ± 10.91	45.34 ± 8.01	0.70
**FAZ**						
Area (mm^2)	.23 ± .10	.35 ± .12	<0.001*	0.24 ± .11	0.34 ± .13	0.003*
Perimeter (mm)	1.88 ± .44	2.38 ± .39	<0.001*	1.92 ± .45	2.31 ± .47	0.003*
Acircularity Index (AI)	1.14 ± .048	1.14 ± .055	0.16	1.15 ± .05	1.14 ± .05	0.21
FD-300 area density (%)	49.20 ± 3.53	51.21 ± 3.76	0.001*	49.92 ± 3.30	51.95 ± 3.54	0.02*
**Choriocapillaris BFA (%)**						
3x3 mm macular area	69.77 ± 3.07	66.10 ± 4.71	0.006*	69.83 ± 2.86	67.29 ± 3.31	0.01*
Fovea	69.95 ± 4.40	66.86 ± 3.77	0.03*	69.72 ± 3.64	67.83 ± 4.35	0.26
Parafovea	69.71 ± 3.94	65.81 ± 3.94	0.008*	69.85 ± 2.87	67.22 ± 3.40	0.01*

^a^P-values were calculated using a mixed-effects linear regression model that adjusted for two eyes of the same patient and hypertension.

^b^P-values were calculated using a mixed-effects linear regression model that adjusted for two eyes of the same patient in a cohort without hypertension.

*Significant at P < 0.05.

HTN: Hypertension; VD: Vessel density, FAZ: Foveal avascular zone, BFA: Blood blow area, SCP: Superficial capillary plexus, ICP: Intermediate capillary plexus, DCP: Deep capillary plexus.

Foveal avascular zone parameters are reported in Table 2. Black subjects had a significantly larger FAZ area (0.35±0.12 vs. 0.23±.10, p<0.001) and FAZ perimeter than White subjects (2.38±0.39 vs. 1.88±0.44, p<0.001). There was no significant difference in the FAZ AI between Black and White subjects (1.14±0.048 vs. 1.14±0.055, p = 0.16). Black subjects had a significantly larger vascular density in a ring with a 300 μm width surrounding the FAZ (FD-300) compared to White subjects (51.21±3.76 vs. 49.20±3.53, p = 0.001). Similar findings were observed in the population of patients without hypertension. Black subjects had a significantly larger FAZ area (p = 0.003), FAZ perimeter (p = 0.003), and FD-300 (p = 0.02) as compared to white subjects without hypertension (Table 2).

Blood flow area in the choriocapillaris is reported in Table 2. Black subjects had lower BFA at the fovea (66.86±3.77 vs. 69.95±4.40, p = 0.03), lower BFA at the parafovea (65.81±3.94 vs. 69.71±3.94, p = 0.008), and lower BFA at the 3x3 mm macular area (66.10±4.71 vs. 69.77±3.07, p = 0.006). In the subgroup of patients without hypertension (Table 2), Black subjects similarly had a significantly lower BFA at the parafovea (p = 0.01) and 3x3 mm macular area (p = 0.01) but not at the area overlying the fovea (p = 0.26).

## Discussion

A wealth of quantitative OCTA studies have emerged in recent years, yet few of these studies have examined differences in OCTA parameters by race or ethnicity. To our knowledge, this study is the first to have analyzed the three retinal capillary plexuses of older Black and White adults with systemic co-morbidities. Our study showed significantly lower foveal VD in the SCP and ICP, along with significantly larger FAZ area, FAZ perimeter, and FD-300 density in Black adults. In the choriocapillaris, Black subjects had decreased BFA across the fovea, parafovea, and 3x3 mm macular area. These findings largely persisted in a subgroup of patients without hypertension.

This study builds off our previous work, which compared the chorioretinal characteristics of young, healthy Black and White adults (average age 27.5 years in Black and 27.8 years in White subjects) [15]. The major elaboration of this study is an analysis of all three retinal capillary plexuses (SCP, ICP, and DCP) and the choriocapillaris layer in older subjects with co-morbidities. While our previous study showed that Black subjects had significantly lower parafoveal VD at the SCP and ICP along with a trend towards lower parafoveal VD at the DCP, we did not observe the same findings herein [15].

Laotaweerungsawat et al. analyzed racial differences in OCTA parameters in a patient population of similar age with adjustments for hypertension and found no difference in the VD at the SCP or DCP between White and Black subjects [18]. Our study also found no differences in parafoveal VD. Advancing age [19,20], hypertension [21–23], smoking [24,25], hyperlipidemia [26], and heart disease [27,28] have all been shown to have deleterious effects on the retinal microvasculature. Despite Black and White subjects being well-matched, it is possible that these systemic factors obscured any parafoveal VD differences that may exist between adults at baseline. However, it is notable that significant differences in FAZ and choriocapillaris parameters were preserved when analyzing a subgroup of patients without hypertension. As noted, hypertension is associated with impaired retinal flow in addition to enlargement of the FAZ [21]. Given that Black subjects had a greater prevalence of hypertension in the overall sample, this may introduce a possible source of confounding. However, our findings in the subgroup analysis suggest that the differences in OCTA parameters were likely not due to this confounding. Furthermore, Black adults within the United States have a higher prevalence of hypertension along with lower rates of blood pressure control as compared to White adults [29]. Additional investigation may be warranted to understand if the increased prevalence of hypertension in Black adults exacerbates baseline differences in FAZ measurements.

Black subjects had lower foveal VD at the SCP and ICP. Consistent with these findings, Black subjects also had greater FAZ area and perimeter, suggesting a diminished capillary network proximal to the FAZ. Numerous previous studies have identified that Black adults have reduced measures of foveal thickness, consistent with deeper foveal pits, as compared to white adults [30–32]. FAZ area has also been shown to correlate negatively with central macular thickness [33,34]. Hence, our findings agree with these prior studies of foveal morphology, as we would expect a thinner fovea to be associated with a larger FAZ area. Interestingly, in a study of infants and children (mean age = 8.5 years), Black pediatric patients had a significantly larger FAZ than white pediatric patients [35]. Hence, the observed differences in FAZ area found herein may be present from birth as opposed to developing throughout the life-course. We also observed a greater FD-300 VD in Black subjects. We propose that this may be a protective, compensatory mechanism that occurs in the juxtafoveal region as a response to a larger baseline FAZ and other differences in foveal structure. Additional investigation of the FD-300 metric is warranted to further understand racial differences in foveal morphology and retinal vascular anatomy.

Interestingly, we did not observe any difference in the FAZ AI between the two racial groups. This suggests that AI may not be subject to variation across race, which we noted with other FAZ parameters. AI is a unitless quotient and does not require correction for axial length, which can otherwise affect FAZ parameters through ocular magnification [36]. The resistance of AI to the effects of ocular magnification and its apparent consistency across race may raise its appeal as a biomarker in studying ocular disease.

Choroidal flow has received increasing attention in OCTA studies due to the vascular supply it provides to the outer retina. In our study, we found that BFA of the choriocapillaris was decreased in Black as compared to White subjects. It is unclear if differences in melanin content in the retinal pigment epithelium (RPE) affect light absorption and subsequent measures of flow in the choriocapillaris. However, prior studies have reported that the melanin content of optic disc melanocytomas did not alter penetration of the OCTA light [37]. Moreover, examination of human autopsy eyes revealed no differences in RPE melanin content with respect to patients of Black and White race [38]. Additionally, interpretation of these results is limited as we did not calculate flow deficits, which may more accurately capture baseline differences in choriocapillaris flow. The average intercapillary area of the choriocapillaris is 5–20 μm while the lateral resolution of the OCTA instrument used in this study is comparably limited to 15–20 μm [39,40]. Hence, we may not have adequately distinguished neighboring capillaries, which should prompt analysis of flow deficits or other textural parameters [41] in the choriocapillaris of different races.

The clinical and epidemiological significance of these findings are unclear and further study of diseased groups are required to elucidate any associations. Various retinal diseases are more prevalent and take on more severe forms in Black adults, including diabetic retinopathy and retinal vein occlusion. Diabetic retinopathy arises in 38.8% of Black adults with diabetes compared to 26.4% of White adults with diabetes [42]. Furthermore, 9.3% of Black adults with diabetes develop vision-threatening diabetic retinopathy, compared with 3.2% of non-Hispanic whites [42]. It is unclear if decreased retinal flow at baseline increases susceptibility to the effects of diabetic microvascular damage, thereby potentially explaining the greater burden of diabetic retinopathy in Black adults. However, FAZ area has been found to increase with progression of diabetic retinopathy severity [43,44], and prospective studies have identified that a larger FAZ area can also predict diabetic retinopathy progression [45,46]. Additionally, one study has found that Black adults have a 58% increased risk of developing a central retinal vein occlusion compared to White adults [47] while Black patients also present with worse visual acuity and develop neovascularization at greater rates in the setting of a retinal vein occlusion [48]. The FAZ area in eyes with retinal vein occlusions is similarly enlarged relative to healthy eyes [49]. Our study was not equipped to directly assess whether an enlarged FAZ at baseline predisposes to greater damage from ischemic insults associated with these retinal conditions. Prospective OCTA studies in patients with diabetes, retinal vein occlusions, and other retinal vascular diseases will better assess the role of race and underlying foveal anatomy as they pertain to epidemiological disparities in the presentations of these conditions.

Limitations of this study include a relatively small patient population from a single clinical site. We selectively recruited our patient cohorts to ensure that they had similar baseline systemic conditions, and we were able to adjust for hypertension given the significant difference between groups. Additionally, we restricted our analysis to Black and White non-Hispanic adults due to these racial groups being well-represented. However, subsequent research should examine OCTA parameters across additional races and ethnicities. Due to our limited patient numbers, we categorized comorbidities as binary variables. Future studies should perform subgroup analysis, exploring variations in extent of hypertension (duration, control, and number of anti-hypertensive medications) and degree of dyslipidemia (as measured by cholesterol levels), among others. While we were not able to adjust for the effect of axial length as this measurement wasn’t available for patients in the study, we did exclude those with high myopia (>5D) or high hyperopia (>3D) where a confounding effect on OCTA parameters is most likely. Finally, we utilized a 3x3 mm field of view, which is relatively small compared to the larger 6x6 mm or even 12x12 mm field of views available on some commercial OCTA machines. However, Kadomoto et al. have reported that OCTA results from a small field of view can predict those from a larger field of view [50,51]. In addition, FAZ parameters have been shown to not vary based on scan size [52]. Strengths include the use of the latest OCTA technology with PAR software, which enabled us to study each of the three capillary plexuses, and well-matched cohorts with respect to systemic comorbidities.

We acknowledge that the use of race in this study is most consistent as a substitute for social constructs and firmly not a representation of biology. Social determinants of health, including but not limited to, access to quality healthcare, environmental living conditions, food insecurity, and educational opportunities are difficult to track in a precise manner but may be underlying factors which explain the results herein. While we did not do so, future studies should examine the interplay between race, social factors, and OCTA parameters.

## Conclusions

This study demonstrated lower foveal and choriocapillaris flow amongst Black subjects with comorbidities as compared to White subjects. While further prospective studies are needed to uncover the full clinical relevance of our findings, our work highlights the importance of establishing diverse normative OCTA databases. Studies of retinal and choroidal diseases using OCTA should attempt to recruit from diverse patient populations whenever possible. Additionally, as OCTA parameters become more common as endpoints in clinical trials, such trials should note the race and ethnicity of subjects, while adjusting for possible effects on quantitative findings in order to avoid confounding.

## Supporting information

S1 Table Raw baseline and OCTA data of subjects included in the study. (XLSX)
